# Gender Disparity in Leading Authorship of Critical Care Clinical Trials: A Systematic Review and Meta-Analysis

**DOI:** 10.7759/cureus.57528

**Published:** 2024-04-03

**Authors:** Sheena Shiwlani, Sanjay Kirshan Kumar, Zubair Rahaman, Yaqub Nadeem Mohammed, Abhi C Lohana, Amit Gulati, Sakshi Khurana

**Affiliations:** 1 Internal Medicine, Mount Sinai Hospital, New York, USA; 2 Gastroenterolgy, Sindh Institute of Urology and Transplantation, Karachi, PAK; 3 Internal Medicine, University at Buffalo Jacobs School of Medicine and Biomedical Sciences, Buffalo, USA; 4 Internal Medicine, Western Michigan University Homer Stryker M.D. School of Medicine, Kalamazoo, USA; 5 Internal Medicine, West Virginia University (WVU) Camden Clark Medical Center, Parkersburg, USA; 6 Cardiology, Icahn School of Medicine at Mount Sinai, New York, USA; 7 Radiology, NewYork-Presbyterian/Columbia University Irving Medical Center, New York, USA

**Keywords:** authorship trends, first authorship, critical care outcomes, gender equity, gender parity

## Abstract

In critical care medicine, research trials serve as crucial avenues for disseminating knowledge, influencing clinical practices, and fostering innovation. Notably, a significant gender imbalance exists within this field, potentially mirrored in the authorship of critical care research. This study aimed to investigate an exploration to ascertain the presence and extent of female representation in first and senior authorship roles within critical care literature. To this end, a systematic search was conducted across PubMed, Google Scholar, and Web of Science databases for original articles published up to February 2024, coupled with a methodological quality assessment via the Newcastle-Ottawa Scale (NOS) and statistical analyses through Review Manager software (RevMan, version 5.4.1, The Cochrane Collaboration, 2020). The study's findings, distilled from seven studies included in the final analysis, reveal a pronounced gender disparity. Specifically, in critical care literature examining mixed populations, female first authors were significantly less common than their male counterparts, with an odds ratio (OR) of 4.25 (95% confidence interval (CI): 3.18-5.68; p < 0.00001). Conversely, pediatric critical care studies did not show a significant difference in gender distribution among first authors (OR: 1.37; 95% CI: 0.31-6.10; p = 0.68). The investigation also highlighted a stark underrepresentation of female senior authors in critical care research across both mixed (OR: 11.67; 95% CI: 7.76-17.56; p < 0.00001) and pediatric populations (OR: 5.41; 95% CI: 1.88-15.56; p = 0.002). These findings underscore the persistent underrepresentation of women in critical care literature authorship and their slow progression into leadership roles, as evidenced by the disproportionately low number of female senior authors.

## Introduction and background

Critical care medicine is a medical field comprising multiple disciplines dedicated to the management of life-threatening conditions and injuries, thus necessitating collaborative efforts among clinicians, researchers, and scientists. In this field, research trials serve as vital conduits for disseminating knowledge, shaping clinical practice, and driving innovation. However, research has revealed a striking imbalance in the gender composition within the critical care field and, subsequently, in the authorship of critical care research. For instance, Holman and colleagues reported that in critical care literature published in 2016, women represented less than 40% of single, first, and senior authors, and this proportion had a negative annual change (-1% per year) [1]. 

Additionally, women also make up a disproportionately small percentage of critical care scholars when it comes to functioning as conference speakers or chairs [2, 3], editorial board members, guideline panelists [4-6], or academic department heads [7]. There are several reasons why this underrepresentation is observed. First, unlike their male counterparts, women are less frequently invited by journals to submit manuscripts and are less likely to secure grants [8, 9]. Additionally, unconscious bias, gender discrimination, and the lack of sufficient role models and mentors all contribute to the underrepresentation of women [10]. Furthermore, fewer women climb the academic hierarchy, which might explain their underrepresentation in scholarly activities.

Given the importance of scientific publications in advancing medical knowledge and developing academic careers, the underrepresentation of female writers in academic medicine is cause for serious concern. Therefore, the current systematic review and meta-analysis aim to meticulously examine existing literature on authorship in the realm of critical care research. This comprehensive evaluation was particularly focused on identifying any potential disparities in authorship roles based on gender. By doing so, the study sought to uncover whether men and women are equally represented as authors in critical care research publications or if there exists a significant imbalance that might suggest underlying gender biases in the field. The objectives of this study were two-fold: firstly, to quantify the representation of genders across authorship positions in critical care research articles, and secondly, to analyze the potential impact of such disparities on the research community and the development of the field. Through a detailed meta-analysis, the study is intended to provide insights into the current state of gender equality in critical care research authorship, contributing to the ongoing discussion on inclusivity and diversity in scientific research.

## Review

Methodology

Literature Search and Information Sources

An in-depth systematic search for scientific literature published from inception until February 2024 was performed on PubMed, Google Scholar, and Web of Science databases. Additionally, bibliographies of articles with similar research objectives as ours were scrutinized for more studies. The search strategy employed in each electronic database was as follows: (Critical care OR Critical care medicine OR Urgent care OR intensive care OR Intensive care unit OR ICU) AND (Authorship OR senior author OR lead author OR first author) AND (gender difference OR sex difference OR female OR women OR men OR female OR Male). Moreover, duplicate articles and unpublished literature were excluded to preserve the scientific integrity and accuracy of our analysis.

Eligibility Criteria

Two impartial reviewers scrutinized the articles retrieved from the electronic databases and included those that matched the criteria listed as follows: 1. articles authored in English. This criterion was explicitly used to avoid direct translations of scientific terms that would have had a negative effect on the scientific nature of the present study; 2. articles that reported the proportion of women occupying the first and senior author positions in critical care literature; 3. articles that included studies of adult or pediatric critical care literature.

On the other hand, studies that did not align with the above criteria or those designed as conference abstracts, editorials, or letters to the editors were excluded. Moreover, studies that did not provide sufficient information on authorship were excluded.

Data Extraction

Two independent reviewers (A.G. and S.K.) carefully examined articles that met our inclusion criteria and collected essential data for in-depth analysis using a standardized form. This data included the first author's surname and publication date (author ID), study design, publication period of the articles, the number of critical care articles reviewed, the total count of authors distinguished by gender, and the proportion of first and senior authors categorized by gender. Disagreements in data collection were resolved through discussions between the reviewers or by consulting an additional (S.S.) reviewer.

Quality Appraisal

A methodological quality evaluation of all included studies was undertaken using the Newcastle-Ottawa Scale (NOS). This technique grades studies based on the selection, comparability, and outcome domains. The selection domain encompassed representativeness of the exposed cohort, selection of the non-exposed cohort, ascertainment of exposure, and demonstration that the outcome of interest was not present at the start of the study. On the other hand, the comparability domain involved evaluating whether the confounders were adjusted for statistical analyses. In contrast, the outcome domain encompassed the assessment of the adequacy of follow-up duration, the ascertainment of outcome data, and whether the follow-up was long enough for the expected outcomes to manifest. For each criterion in the domains mentioned above, a maximum of one star was assigned when the assessment information was well-documented in the studies; otherwise, no star was assigned.

Data Synthesis

All statistical analyses in our study were performed using the Review Manager software (RevMan version 5.4.1, The Cochrane Collaboration, 2020). Since all the data were dichotomous, the overall effect size was calculated using the simple odds ratio (OR) and its corresponding 95% confidence interval (CI). A random effects model was employed to estimate effect sizes because it provides more conservative outcomes. Additionally, the interstudy heterogeneity was calculated using the I2 statistics, of which values greater than 75%, between 50 and 75%, and less than 50% indicated high, moderate, and low degrees of heterogeneity, respectively. Whenever possible, subgroup analyses were conducted according to the population studied in each critical care research project (i.e., mixed or pediatric). 

Results

Study Selection

**Figure 1:**
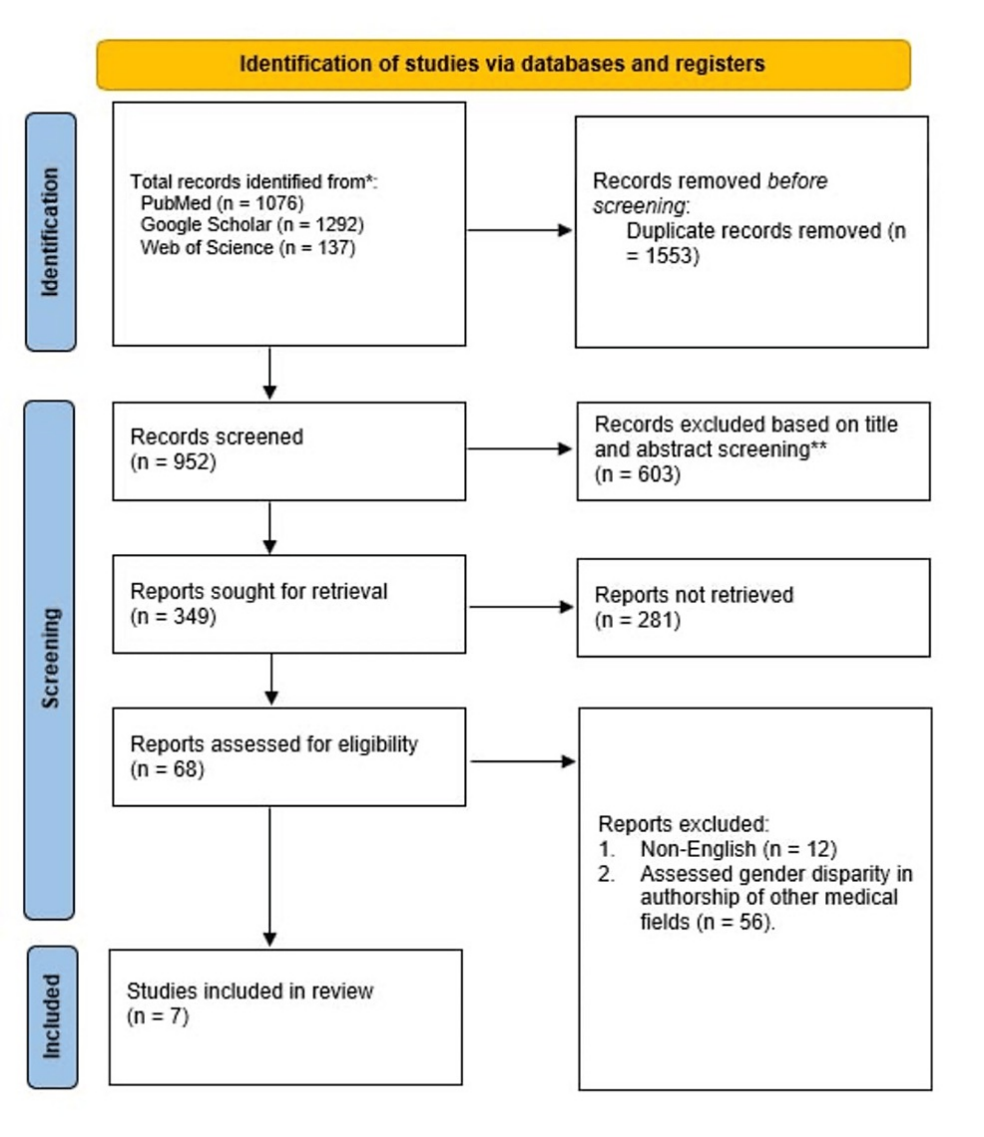
A PRISMA flow diagram outlining the study selection process PRISMA: Preferred Reporting Items for Systematic Reviews and Meta-Analyses

After the in-depth database search, we identified 2,505 records with the predefined Medical Subject Headings (MeSH) phrases. A thorough inspection of these records led to the elimination of 1,553 records that were deemed identical. Of the remaining 952 distinct records, 603 were eliminated after being deemed to have irrelevant titles and abstracts. Moreover, we did not retrieve 281 records because they were conference abstracts, editorials, or conference abstracts. The remaining records were assessed according to the eligibility criteria, of which only seven met the inclusion criteria. The other 68 records were excluded primarily because 12 of them were published in different languages, and the remaining 56 compared gender disparity in the authorship of other medical fields. The full selection criteria are outlined in Figure 1.

Study Characteristics

Seven studies were included in the final analysis. These studies assessed gender differences in the authorship of 29,043 pieces of critical care literature. There were 20,512 first authors and 16,192 senior authors whose gender could be identified. Furthermore, the study periods for the assessed critical care research articles ranged from 1986 to 2022 (Table 1).

**Table 1:** Summary of the study characteristics The data have been represented in percentages.

Author ID	Study design	Study period	No. of articles reviewed	Total no. of authors		First author, n (%)		Senior author, n (%)	
				First authors	Senior authors	Male	Female	Male	Female
Chander et al., 2023 [11]	Retrospective bibliometric study	2000 – 2022	1,398	1,398	1,398	1,054 (75.4)	344 (24.6)	1,166 (83.4)	232 (16.6)
Chary et al., 2021 [12]	Retrospective bibliometric study	2008 – 2018	7,370	5,153	3,890	3,396 (65.9)	1,757 (34.1)	3,128 (80.4)	762 (19.6)
Gershengorn et al., 2022 [13]	Bibliometric study	2018 - 2020	721	676	687	461 (68.2)	215 (31.8)	531 (77.3)	156 (22.7)
Jeyapalan et al., 2023 [14]	Bibliometric study	2002 – 2021	302	302	283	134 (44.4)	168 (55.6)	181 (60)	102 (36)
Mehta et al., 2022 [15]	Quantitative content analysis	1994 – 2020	354	354	354	212 (59.9)	142 (40.1)	235 (66)	119 (34)
Vranas et al., 2020 [16]	Bibliometric study	2008 – 2018	18,483	12,244	9,204	8,035 (65.6)	4,209 (34.4)	7,027 (76.3)	2,177 (23.7)
Xu et al., 2020 [17]	Bibliometric study	1986 – 2018	415	385	376	243 (63)	142 (37)	283 (75)	93 (25)

Study Quality

A summary of the quality evaluation using NOS is presented in Table 2. Out of the seven studies, four had fair quality (NOS score: 6-7) and three had poor quality (NOS score: ≤5). The most common reason for reduced methodological quality was poor comparability, as most studies only correlated gender with first and senior authorship.

**Table 2:** Methodological quality evaluation using the Newcastle-Ottawa Scale

Study	Selection (/4)	Comparability (/2)	Outcome (/3)	Overall methodological quality
Chander et al., 2023 [11]	3	2	2	Fair
Charv et al.,2021 [12]	3	l	2	Fair
Gershengom et al., 2022 [13]	3	l	l	Poor
Jeyapalan et al., 2023 [14]	2	l	2	Poor
Mehta et al., 2022 [15]	3	l	2	Fair
Vranas et al., 2020 [16]	3	l	2	Fair
Xu et al., 2020 [17]	2	l	2	Poor

Gender Difference in First Authorship

**Figure 2:**
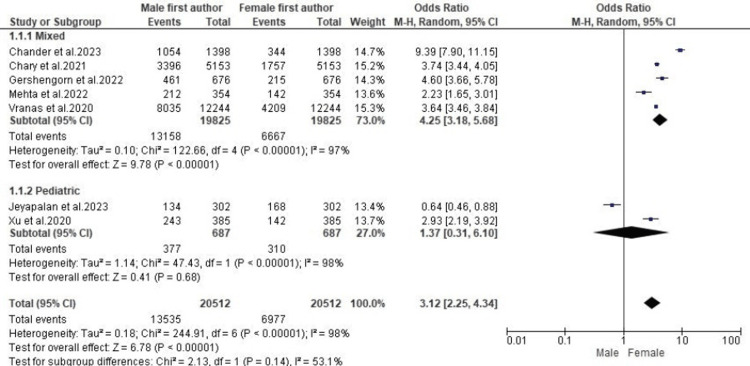
A forest plot showing gender differences in first authorship OR: odds ratio; CI: confidence interval; M-H: Mantel–Haenszel [11-17]

Out of the seven included studies, five reported gender differences in the authorship of critical care research of mixed populations, while the other two reported gender differences in the research of pediatric patients only. A subgroup analysis of data from these studies showed that the proportion of female first authors of critical care research involving a mixed population was significantly less compared with male first authors (OR: 4.25; 95% CI: 3.18-5.68; p < 0.00001) (Figure 2). However, the proportion of female first authors did not differ significantly from male first authors of pediatric critical care research (OR: 1.37; 95% CI: 0.31-6.10; p = 0.68) (Figure 2). Further statistical analysis showed that a high level of heterogeneity persisted in all subgroups.

Gender Difference in Senior Authorship

**Figure 3:**
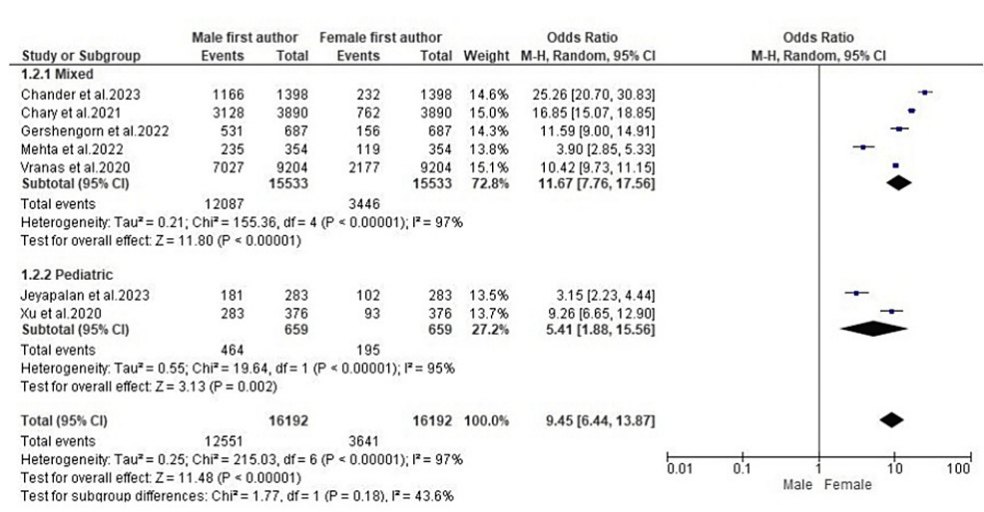
A forest plot showing gender differences in senior authorship OR: odds ratio; CI: confidence interval; M-H: Mantel–Haenszel [11-17]

A subgroup analysis of data from the seven included studies showed that the proportion of female senior authors was significantly lower compared to male first authors of critical care literature with mixed populations (OR: 11.67; 95% CI: 7.76-17.56; p < 0.00001) (Figure 3). Similarly, the subgroup analysis showed that the proportion of female senior authors was significantly lower than that of male senior authors of pediatric critical care research (OR: 5.41; 95% CI: 1.88-15.56; p = 0.002) (Figure 3). However, the heterogeneity of all subgroup analyses was substantial.

Trends in Gender Differences

Due to inconsistencies in how the data regarding the percentage of female first and senior authors throughout various eras were recorded, we could only do a qualitative data analysis. Mehta and colleagues found that for studies published between 1994 and October 2020, the percentage of women occupying the first author position ascended from 21% between 1994 and 2000 to 31% between 2001 and 2005, to 32% between 2006 and 2010, to 38% between 2011 and 2015, after which the rate stagnated until 2020. On the other hand, they found that the proportion of senior female authors declined from 15% between 1994 and 2000 to 4% between 2001 and 2005; afterward, the number of senior female authors increased to 29% between 2006 and 2010, 37% between 2011 and 2015, and 42% between 2016 and 2020 [15]. Similarly, Vranas and colleagues found that the proportion of female first authors increased from 27.5% in 2008 to 32.6% in 2018, at an annual increase of 0.44% (p<0.01), while the average yearly increase in female senior authors during the same period was 0.51% (p<0.01) [16]. In contrast, Chary et al. [12]. found that the proportion of female first and senior authors stayed the same between 2008 and 2018. Another study investigated how COVID-19 impacted the authorship of critical care research and found no considerable difference in the proportion of female first and senior authors during the COVID-19 and post-COVID-19 eras [13].

In addition, one of the studies investigating gender differences in the authorship of pediatric critical care literature found that the number of female senior authors had increased significantly over the years (from 0% between 1985 and 1989 to 33% between 2015 and 2018; p = 0.004). The proportion of female first authors increased; however, the difference was statistically insignificant (17% between 1985 and 1989 to 39% between 2015 and 2018; p = 0.22) [17]. On the other hand, a study investigating gender differences in the authorship of publications from Pediatric Acute Lung Injury and Sepsis Investigators (PALISI) reported that the number of female first authors between 2012 and 2021 was considerably higher compared to the years between 2002 and 2011 (60.2% vs. 37.7%; p = 0.002). However, the proportion of female senior authors did not change over time [14].

Discussion

In the past half-century, the percentage of women joining medical schools has grown drastically [18]. Specifically, the Association of Medical Colleges claimed that as of 2005, female medical students in the US alone were approximately 49% of the total enrollment, which is a significant increase from the 6% recorded in 1960 [18]. Likewise, in the United Kingdom, women constituted 59% of all annual admissions to medical faculties in 2009, as opposed to 32% in 1977 [19]. However, despite this considerable increment, our study found that the proportion of female first and senior authors is significantly low compared to male first and senior authors. This finding is consistent with what has been reported in other studies. For instance, out of 27.3 million researchers in 5.5 million scientific journals published via the Web of Science between 2008 and 2012, over one-third were men [20]. Similarly, research conducted in 2016 revealed that while the percentage of women occupying the first author's position in journals with substantial impact rose from 27% in 1994 to 37% in 2014, the number had stagnated since 2000, suggesting that women are still underrepresented in high-impact journals [21,22].

Several factors might explain the gender gap reported in our meta-analysis. First, the proportion of female faculty members capable of serving as senior authors and editorial commentators remains limited. For instance, Nonnemaker investigated the academic advancements of men and women in different medical school faculties in the United States between 1979 and 1997 and found that the proportion of women who advanced into associate and full professor ranks was considerably lower than anticipated [23]. Similarly, data from the American Medical Colleges showed that by 2004, women accounted for 19% of all associate and full professors [24]. Due to this low pool, women occupying senior positions may be overwhelmed with academic activities and find it necessary to decline invitations for publication more often, despite the potential for prestige and influence. Secondly, the gender disparity might have stemmed from the career choices of men and women. Research has shown that male and female medical students have extremely diverse career choices [25,26], with women far more inclined to spend their time engaging in educational and clinical activities than taking part in research activities [27]. Furthermore, while studies indicate that work-life balance is equally valued by men and women, particularly among younger physicians, other research suggests that women may prioritize the balance between work and personal activities differently. Specifically, Nonnemaker reported that fewer women were inclined to select academic career paths in the late 1990s [23].

Thirdly, the “confidence disparity” between men and women reported in other professions might have played a role in women's hesitation to hand in their critical care articles. A Hewlett-Packard survey found that female employees only felt the need to apply for promotions when they were certain that they fulfilled all the required credentials for a specific role, whereas their male counterparts sought the exact same position when they were certain they exceeded only 60% of the credentials [28]. Similarly, a 2017 study investigated gender differences in papers published in science in 2015 and found that less than one-third of female authors submitted to journals of their respective fields, which suggests a confidence issue as this proportion was lower than anticipated [29]. Fourth, there is growing evidence that female researchers tend to have limited access to mentoring, networking, or funding opportunities, which is further compounded by their higher work-life commitment and patriarchal organizational setup [30,31]. For instance, recent studies have shown that men were more likely to spend their time in research, service, and administrative roles, while women had a higher teaching load, which restricted their research load [31,32]. Moreover, a 2021 study suggested that women applied for fewer grants than men, which might hinder their research activity [33]. Finally, the underrepresentation of women reported in our study might have stemmed from the fact that first-time female authors tend to submit their research articles to low-impact journals.

While our findings have shown a gender imbalance in the authorship of critical care research, we found no significant difference between female and male first authors of studies focused on pediatric critical care. This finding may be attributed to women dominating the pediatric medical field [34]. However, the proportion of female senior authors was significantly low compared to male senior authors. This shows that despite the high number of women in pediatrics, those who advance to senior positions are relatively few. For example, a 2018 research in the United States showed that despite women residents being more than 70%, this proportion decreased over the subsequent career steps, with only 27.5% advancing to department chair positions [34]. Moreover, the low proportion of women in senior authorship might be explained by the fact that many young women entering the academic pediatric field often exit the scientific career path earlier than men [35], a phenomenon referred to as a leaking pipeline [36]. This early exit might be due to the fact that female graduate students are less likely to aspire for leadership positions due to different life priorities such as parenthood [36], caring for the family [37], or a satisfying life-work balance [38], but also due to limited role models [39].

The trend of increasing female first and senior authors signifies progress towards gender equality in academia and research. It reflects greater inclusivity and diversity in leadership roles within the scientific community, encouraging innovation and providing role models for future female researchers. However, this increment is minimal, suggesting the need for strategies to overcome gender imbalances in the authorship of critical care literature. Several approaches have been employed to overcome this gender imbalance. For example, the University of California, Davis School of Medicine, came up with a program to offset the challenges experienced by women during career progression. This initiative encompassed specific advocacy for women's progression in positions of authority and gathered workforce information to enable organizations to make educated choices and take definitive actions. Within the initial 10 years of executing this initiative, the percentage of female instructors doubled, with a notable rise in the proportion of full-time professors and departmental chairs [40]. Similarly, the National Institutes of Health's (NIH) Office of Research on Women’s Health has launched additional effective programs that encourage the career progression of women in the biomedical sciences. These initiatives comprise programs that encourage researchers to return to the profession after a qualifying hiatus and the NIH Working Group on Women in Biomedical Careers, which aids with mentorship and networking opportunities [41]. Furthermore, prominent journals have made advances to address gender inequalities in academic publications by establishing diversity objectives for commissioned material, peer reviewers, and editorial responsibilities, as well as educating editors on diversity efforts and unconscious gender bias [42, 43].

Limitations

The current is subject to several limitations that ought to be accounted for during the interpretation of the findings. First, our statistical analyses have shown that high interstudy heterogeneity persists in all subgroup analyses. This heterogeneity probably resulted from the variation in sample sizes and study periods for the analyzed studies. Secondly, it is possible that the included studies did not identify all critical care research published between the periods they specified, therefore introducing selection bias in their research and subsequently undermining our statistical analyses. Thirdly, there is a risk of gender misclassifications because, in all the studies, gender was not self-reported by the authors but rather classified based on their names. This misclassification might have undermined the proportion of men and women occupying the first and senior author positions. Fourth, since all the studies analyzed male and female researchers only, those who identify as non-binary, trans, or non-conformity individuals could not be assessed in the present meta-analysis. Fifth, due to variations in how the data for authorship were reported over different time periods, we could only carry out a qualitative data review. Finally, despite providing reasons why the gender gap was observed in critical care research, these reasons were entirely speculative because none of the studies evaluated possible factors associated with the gender gap in authorship. Therefore, future studies should strive to explore these factors, as it is the only way we can definitively conclude why gender imbalance is observed in critical care research.

## Conclusions

In summary, our study suggests that despite substantial strides made by women in the medical field over the past five decades, a gender imbalance still exists in the authorship of high-impact journals, especially in the critical care field. Furthermore, our investigation has shown that women in critical care medicine are slow to advance into leadership positions. Therefore, further investigation is required to fully understand why gender imbalance exists in the authorship of critical care literature and to determine possible barriers that impede women from advancing into senior positions, thus limiting their establishment into prominent authorship positions. Addressing gender imbalances in critical care literature is vital for achieving an equitable scientific community. Investigating the causes can guide targeted actions like mentorship for female researchers and fair authorship policies, enriching research with diverse perspectives, and enhancing scientific quality.
